# Fault Diagnosis for Rolling Bearings Based on Fine-Sorted Dispersion Entropy and SVM Optimized with Mutation SCA-PSO

**DOI:** 10.3390/e21040404

**Published:** 2019-04-16

**Authors:** Wenlong Fu, Jiawen Tan, Yanhe Xu, Kai Wang, Tie Chen

**Affiliations:** 1 College of Electrical Engineering & New Energy, China Three Gorges University, Yichang 443002, China; 2 Hubei Provincial Key Laboratory for Operation and Control of Cascaded Hydropower Station, China Three Gorges University, Yichang 443002, China; 3 School of Hydropower and Information Engineering, Huazhong University of Science and Technology, Wuhan 430074, China

**Keywords:** rolling bearings, fault diagnosis, variational mode decomposition, fine-sorted dispersion entropy, mutation sine cosine algorithm-particle swarm optimization, support vector machine

## Abstract

Rolling bearings are a vital and widely used component in modern industry, relating to the production efficiency and remaining life of a device. An effective and robust fault diagnosis method for rolling bearings can reduce the downtime caused by unexpected failures. Thus, a novel fault diagnosis method for rolling bearings by fine-sorted dispersion entropy and mutation sine cosine algorithm and particle swarm optimization (SCA-PSO) optimized support vector machine (SVM) is presented to diagnose a fault of various sizes, locations and motor loads. Vibration signals collected from different types of faults are firstly decomposed by variational mode decomposition (VMD) into sets of intrinsic mode functions (IMFs), where the decomposing mode number *K* is determined by the central frequency observation method, thus, to weaken the non-stationarity of original signals. Later, the improved fine-sorted dispersion entropy (FSDE) is proposed to enhance the perception for relationship information between neighboring elements and then employed to construct the feature vectors of different fault samples. Afterward, a hybrid optimization strategy combining advantages of mutation operator, sine cosine algorithm and particle swarm optimization (MSCAPSO) is proposed to optimize the SVM model. The optimal SVM model is subsequently applied to realize the pattern recognition for different fault samples. The superiority of the proposed method is assessed through multiple contrastive experiments. Result analysis indicates that the proposed method achieves better precision and stability over some relevant methods, whereupon it is promising in the field of fault diagnosis for rolling bearings.

## 1. Introduction

Rolling bearings are a crucial part in modern industrial manufacture, which can be found in linear guides, precision machine tools, and engine parts, etc., whose failure may result in serious safety accidents and economic loss. Therefore, one of the major topics to be investigated in preventing failures of mechanical systems is recognizing and diagnosing rolling bearing faults [1,2]. Unfortunately, rolling bearings are prone to failure to some extent because of complex operating conditions and structural characteristics [3]. Given this fact, effective and feasible fault diagnosis methods need to be developed for rolling bearings, thus, to improve the reliability of mechanical systems [4].

Due to the rich information carried by vibration signal, most of fault diagnosis methods for rolling bearings rely on analyzing vibration signal [5]. However, the vibration signal is commonly non-stationary in actual operation, which restricts the extraction efficiency for fault features and affects the accuracy of fault diagnosis. Addressing this issue, many time-frequency signal analysis methods have been proposed to analyze non-stationary signals, including wavelet transform (WT), empirical mode decomposition (EMD), ensemble empirical mode decomposition (EEMD), and variational mode decomposition (VMD). WT is a self-adaptive signal decomposition method, which has a good frequency resolution and time resolution for low-frequency components and provides a good frequency resolution and time resolution for high-frequency components [6], but the selection of an appropriate wavelet filter bank remains a challenge. EMD is adept in dealing with non-stationary signals and does not need to pre-set any basis function [7], but its performance is affected by end effects and mode mixing. To overcome these defects, as an improved version of EMD, EEMD was proposed by introducing noise-assisted analysis method [8], but it also increases the computational cost and cannot completely neutralize the added noise. Unlike the methods mentioned above, VMD is a quasi-orthogonal signal decomposition method, the main structure of which is to construct and solve a constrained variational problem [9]. Hence it realizes the separation of the frequency of each signal component, avoiding the problem of mode mixing in EMD and EEMD or filter selection in WT. Its advancement and effectiveness have been demonstrated in many previous studies. For instance, Fu et al. [10] applied VMD to decompose the wind speed time series into a set of sub-series with various frequency scales and then calculated the final predicted value of the raw wind speed by accumulating the predicted results of all components. Li et al. [11] established the approximate entropy feature vectors for each component of VMD and obtained a good fault diagnosis effect for rolling bearings. Fu et al. [12] achieved the feature extraction of signals by conducting multivariate autoregressive modelling for components of VMD.

As the non-stationarity of signals weakened by signal analysis methods, fault features can be smoothly extracted. Entropy, as a feature extraction method based on non-linear dynamic parameters, is widely applied in medical and mechanical fields, which can reflect the complexity of time series and is an effective tool for analysis of non-stationary time series. Approximate entropy (ApEn) [13] is the pioneer of introducing a feature extraction algorithm from the medical field into mechanical fault diagnosis, while it is susceptible to outliers. Permutation entropy (PE) [14] has strong anti-noise ability and is simple in calculation, but it does not take into account the difference between the average amplitude and actual amplitude. Fuzzy entropy (FE) [15] introduces the concept of uncertainty reasoning to solve the defect of sample entropy [16] which adopts a hard threshold as a discriminant criterion which may result in unstable results. This approach, however, also lowers the computational efficiency. Recently, a novel irregularity indicator termed dispersion entropy (DE), was proposed by Mostafa and Hamed [17]. DE has the advantages of fast calculation speed and less influence from outliers, which overcomes the defects of ApEn and PE to some extent, but the relationship information between neighboring amplitudes is not considered enough. In this paper, a concept of fine-sorted dispersion entropy (FSDE) is proposed to measure the features of vibration data and then construct fault feature vectors. By introducing an additional factor, FSDE is more perceptive to element values and may have better feature extraction ability than DE.

The essence of fault diagnosis for rolling bearings is actually a pattern recognition problem, that is, fault type is determined by the fault features of samples. In an engineering application, there are many alternative methods available for solving this problem. For instance, Bayesian decision has strong recognition ability by considering prior probability as well as class conditional probability, and its good accuracy requires the assumption of a suitable prior model [18]. K-nearest neighbor (KNN) based on Euclidean or Manhattan distances is easy to realize and susceptible to the distribution of samples [19]. Artificial neural network (ANN) has good ability in processing recognition problem with the support of a large number of samples [20]. Based on statistical learning and structural risk minimization theory, support vector machine (SVM) is an excellent machine learning method, which finds an optimal hyperplane that meets the classification requirements. The hyperplane maximizes the interval between two classes while promoting classification performance. Currently, SVM combined with the feature extraction method has been successfully employed in the field of pattern recognition [21,22].

Although SVM possesses superior ability in pattern recognition, its performance is affected by parameters. In view of this, various optimization algorithms have been developed and applied to search the best parameters, such as particle swarm optimization (PSO) [23], bacterial foraging algorithm (BFA) [24], artificial sheep algorithm (ASA) [25] and sine cosine algorithm (SCA) [26]. As a novel optimization approach, the effectiveness of SCA in parameter optimization has been proved in many previous studies [27,28]. To achieve better performance in convergence precision, a hybrid optimization strategy combining mutation operator, sine cosine algorithm, and particle swarm optimization (MSCAPSO) is developed in this paper. VMD was firstly applied to decompose the vibration signal into a set of intrinsic mode functions (IMFs), and the decomposing mode number *K* was pre-set with central frequency observation method. Then a modified dispersion entropy containing the additional factor (FSDE) is proposed to construct the feature vectors of IMFs for different types of faults. Afterward, a hybrid optimization strategy combining mutation operator, SCA and PSO called MSCAPSO was proposed for parameter selection of SVM. Finally, the optimal SVM model was employed to classify different fault samples. The engineering application and contrastive analysis reveal the availability and superiority of the proposed method.

The paper is organized as follows: Section 2 is dedicated to the basic knowledge of VMD and SVM. Section 3 introduces the proposed fault diagnosis method based on fine-sorted dispersion entropy and MSCA-PSO optimization approach. Section 4 presents the engineering application, the result analysis of which demonstrates the superiority of the proposed method. Some discussion about the method presented in this paper is in Section 5. The conclusion is summarized in Section 6.

## 2. Fundamental Theories

### 2.1. Variational Mode Decomposition

Variational mode decomposition (VMD) is a new adaptive signal preprocessing method [29]. By setting a mode number *K* in advance, the given signal can be decomposed into *K* band-limited intrinsic mode functions (IMFs). The cyclic iterative method is used to obtain the optimal solution of the constrained variational problem, through which the frequency center and bandwidth of each mode are determined. The constrained variation problem can be described as follows:

(1) $$\begin{array}{l} \underset{m_{k},\omega_{k}}{\min}\{\sum_{k}{\left\|\partial_{t}\left[\left(\delta (t)+\frac{j}{\pi t}\right)*m_{k}(t)\right]e^{-j\omega_{k}t}\right\|}_{2}^{2}\}\\ s.t.\;\sum_{k=1}^{K}m_{k}(t)=f(t),\;k=1,2,\ldots ,K \end{array}$$

where *m_k_* = [*m*_1_, *m*_2_, …, *m_k_*] represents the set of *K* mode functions and $\omega_{k}=[\omega_{1},\omega_{2},\ldots ,\omega_{k}]$ represents the set of central frequencies, while *∂_t_* and *δ_t_* are the partial derivative of time *t* for the function and unit pulse function, respectively. *f*(*t*) is the given real valued input signal.

For the above variational problem, quadratic penalty function term and Lagrange multiplier are employed to transform it into an unconstrained problem. Then Problem (1) can be specified as follows:

(2) $$\begin{array}{ll} L(m_{k},\omega_{k},\beta ) & =\alpha \sum_{k}{\left\|\partial_{t}\left[\left(\delta (t)+\frac{j}{\pi t}\right)*m_{k}(t)\right]e^{-j\omega_{k}t}\right\|}_{2}^{2}+\\ & {\left\|f(t)-\sum_{k}m_{k}(t)\right\|}_{2}^{2}+\langle \beta (t),f(t)-\sum_{k}m_{k}(t)\rangle \end{array}$$

where *α* and *β*(*t*) represent the penalty factor and Lagrange multiplier, respectively.

Then alternate direction method is utilized to obtain the saddle point of Lagrange multiplier, that is, solving (2) by optimizing *m_k_*, *ω_k_*, and *β* alternately. The optimization problems of *m_k_* and *ω_k_* are formulated as (3) and (4), respectively.

(3) $$m_{k}^{n+1}=\min\left\{\alpha {\left\|\partial_{t}\left[\left(\delta (t)+\frac{j}{\pi t}\right)*m_{k}(t)\right]e^{-j\omega_{k}t}\right\|}_{2}^{2}+{\left\|f(t)-\sum_{i}m_{i}(t)+\frac{\beta (t)}{2}\right\|}_{2}^{2}\right\}$$

(4) $$\omega_{k}^{n+1}=\min\left\{{\left\|\partial_{t}\left[\left(\delta (t)+\frac{j}{\pi t}\right)*m_{k}(t)\right]e^{-j\omega_{k}t}\right\|}_{2}^{2}\right\}$$

Solving problems (3) and (4), the iterative equations are deduced as follows:

(5) $${\hat{m}}_{k}^{n+1}(\omega )=\frac{\hat{f}(\omega )-\sum_{i\neq k}{\hat{m}}_{i}(\omega )+\frac{\hat{\beta }(\omega )}{2}}{1+2\alpha {\left(\omega -\omega_{k}\right)}^{2}}$$

(6) $$\omega_{k}^{n+1}=\frac{\int_{0}^{\infty }\omega {\left|{\hat{m}}_{k}(\omega )\right|}^{2}d\omega }{\int_{0}^{\infty }{\left|{\hat{m}}_{k}(\omega )\right|}^{2}d\omega }$$

The Lagrange multipliers can be iterated with Equation (7).

(7) $${\hat{\beta }}^{n+1}(\omega )={\hat{\beta }}^{n}(\omega )+\gamma (f(\omega )-\sum_{k}{\hat{m}}_{k}^{n+1}(\omega ))$$

where *γ* is an updating parameter.

The main steps of VMD can be summarized as follows:

*Step 1*: Initialize *m_k_*^1^, *ω_k_*^1^, *β*^1^, *n* = 1;

*Step 2*: Execute loop, *n* = *n* + 1;

*Step 3*: Update *m_k_* and *ω_k_* based on Equations (5) and (6);

*Step 4*: Update *β* based on Equation (7);

*Step 5*: If $\sum_{k}{\left\|{\hat{m}}_{k}^{n+1}-{\hat{m}}_{k}^{n}\right\|}_{2}^{2}/{\left\|{\hat{m}}_{k}^{n}\right\|}_{2}^{2}<\varepsilon$ loop end, else turn to *step* 2 for next iteration.

### 2.2. Support Vector Machine

Support vector machine (SVM) is a machine learning model developed by Vapnik [30], which can deduce the optimal solution between model complexity and learning ability based on limited information, thus, to obtain the best classification accuracy, showing unique advantages in solving learning problems with limited samples, non-linear, and high dimensional data. For a given sample set $\left\{\left(x_{i},y_{i}\right)|i=1,\;2,\ldots ,n\right\}$, the main idea of SVM is to map samples into a high-dimensional feature space and find a hyper-plane to solve the corresponding non-linear problem in low-dimensional space. The hyper-plane function can be constructed as:

(8) w⋅x+b=0

where *w* is a weight vector, and *b* is a bias parameter, while *w*·*x* represents the inner product of *w* and *x*.

Taking binary classification issue as an example, to classify samples correctly with enough into classification intervals, all samples are required to meet the following constraint:

(9) $$w\cdot x_{i}+b\left\{ \begin{array}{l} >1\;for\;y_{i}=1\\ <-1\;for\;y_{i}=-1 \end{array}\right.$$

In the sequel, the classification interval can be calculated as 2/‖*w*‖^2^, and maximizing the interval is equivalent to minimizing ‖*w*‖^2^. To solve the linear indivisibility problem, the slack term *ξ* and penalty factor *C* are brought into Equation (9), hence the construction of the optimal hyper-plane is transformed into a constrained optimization problem:

(10) $$\left\{ \begin{array}{l} \min f=\frac{1}{2}{\left\|w\right\|}^{2}+C\sum_{i=1}^{n}\xi_{i}\\ s.t.\;y_{i}({\vec{w}}^{T}\cdot x_{i}+b)\geq 1-\xi_{i},\;i=1,2,\ldots ,n \end{array}\right.$$

When mapping samples to high-dimensional space, different inner product kernel functions will form different algorithms. Among them, radial basis function is widely employed in the application of pattern recognition, which can be described as:

(11) $$K(x_{i},x_{j})=\phi (x_{i})\cdot \phi (x_{j})=\exp(-g\|x_{i}-x_{j}{\|}^{2})$$

where *g* is a kernel parameter.

The solution of the constrained optimization problem (10) is determined by a saddle point of Lagrange function, and this quadratic programming problem is transformed into the corresponding dual problem, that is:

(12) $$\begin{array}{l} \max L=\sum_{i=1}^{n}\mu_{i}-\frac{1}{2}\sum_{i,j=1}^{n}\mu_{i}\mu_{j}y_{i}y_{j}K(x_{i},x_{j})\\ s.t.\;\sum_{i=1}^{n}\mu_{i}y_{i}=0,\;\mu_{i}\geq 0,\;i=1,2,\ldots ,n \end{array}$$

where *µ_i_* is Lagrange multiplier.

With the optimal value of *µ_i_* obtained from the above dual problem, the optimal classification discriminant function can be ascertained as:

(13) $$f(x)=sgn(\sum_{i=1}^{n}\mu_{i}K(x_{i},x)+b)$$

## 3. Fault Diagnosis for Rolling Bearings by Fine-sorted Dispersion Entropy and Mutation SCA-PSO Optimized SVM

### 3.1. Fine-Sorted Dispersion Entropy

#### 3.1.1. Dispersion Entropy

For a time series ***x*** = [*x*_1_, *x*_2_, …, *x_n_*] of length *n*, *x_i_* is firstly normalized by a mapping function, where the standard normal cumulative distribution function is generally used [17].

(14) $$y_{i}=\frac{1}{\sigma \sqrt{2\pi }}\int_{-\infty }^{x_{i}}e^{\frac{-{\left(s-\mu \right)}^{2}}{2\sigma^{2}}}ds$$

where *i* = 1, 2, …, *n*, *σ*, and *μ* are the variance and expectation of the normal distribution, respectively. The time series *x* is then normalized to ***y*** = [*y*_1_, *y*_2_, …, *y_n_*], *y_i_* ∈ (0, 1). Further, the phase space is reconstructed for ***y***:

(15) $$y_{j}^{m}=[y_{j},y_{j+\tau },\ldots ,y_{j+(m-1)\tau }]$$

where *j* = 1, 2, …, *n* − (*m* − 1)*τ*, *m* and *τ* are the embedding dimension and time delay, respectively. Then $y_{j}^{m}$ is mapped to *c* class (1 to *c*):

(16) $$z_{i}^{c}=round(c\cdot y_{i}+0.5)$$

(17) $$z_{j}^{m,c}=[z_{j}^{c},z_{j+\tau }^{c},\ldots ,z_{j+(m-1)\tau }^{c}]$$

where $z_{i}^{c}$ is the *i*-th element of class sequence ***z**^c^*, while *round* means rounding. Each $z_{j}^{m,c}$ corresponds to a dispersion pattern $\pi_{v_{0}v_{1}\ldots v_{m-1}}$ with elements $z_{j}^{c}=v_{0},\;z_{j+d}^{c}=v_{1},\ldots ,\;z_{j+(m-1)\tau }^{c}=v_{m-1}$. Since $\pi_{v_{0}v_{1}\ldots v_{m-1}}$ contains *m* elements, each element contains *c* values, so the number of all possible dispersion patterns are *c^m^*.

The relative frequency of $\pi_{v_{0}v_{1}\ldots v_{m-1}}$ can be deduced as:

(18) $$p=\frac{Number\{j|j\leq n-(m-1)\tau ,\pi_{v_{0}v_{1}\ldots v_{m-1}}\}}{n-(m-1)\tau }$$

where $Number\{j|j\leq n-(m-1)\tau ,\pi_{v_{0}v_{1}\ldots v_{m-1}}\}$ is the number of occurrences of each $\pi_{v_{0}v_{1}\ldots v_{m-1}}$ corresponding to $z_{j}^{m,c}$:

Dispersion entropy can be defined according to information entropy theory:

(19) $$DE(x,m,c,\tau )=-\sum_{\pi =1}^{c^{m}}p\cdot \ln(p)$$

A large DE value means that the time series is more irregular; on the contrary, the smaller the DE value, the lower the irregularity.

#### 3.1.2. Fine-Sorted Dispersion Entropy

**Figure 1:**
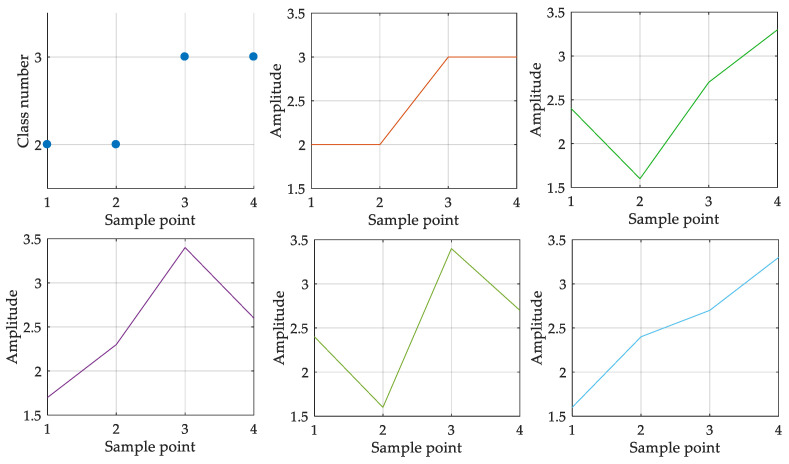
Example of possible vectors corresponding to the same dispersion pattern.

Dispersion entropy, as an information entropy feature, can effectively monitor the dynamic changes in vibration signal and measure its irregularity, so it can be effectively applied to the state monitoring of rolling bearings. However, its ability to feature extraction for the complex non-stationary signal can be further improved in some respects. One possible improvement would be to enhance the perception of neighboring element values. In the theory of dispersion entropy, dispersion entropy focuses on the class sequence that mapping the elements of time series into positive integers. The relationship between elements, as comparatively critical information, has not been considered enough, which suggests that the degree of difference between neighboring elements is not concerned. According to the mapping rule of dispersion entropy, the same dispersion pattern may come from multiple forms of sample vectors. Figure 1 is an example of possible vectors corresponding to the same dispersion pattern (dispersion pattern [2 2 3 3]).

**Figure 2:**
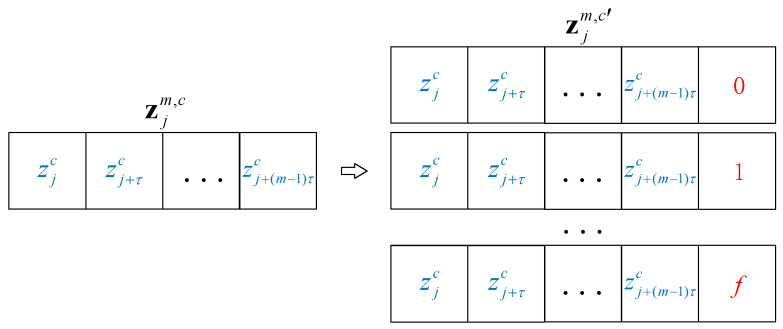
*f* is added in class vector of dispersion entropy as an additional element.

To enhance the feature extraction ability of dispersion entropy, an improved dispersion entropy called fine-sorted dispersion entropy (FSDE) is proposed. FSDE introduces a factor *f* to measure the difference between neighboring elements in the vector $y_{j}^{m}$. The factor *f* is then added in class sequence $z_{j}^{m,c}$ as an additional element. Therefore, the relationship information between elements is brought into dispersion entropy. This operation not only inherits the features within the result of original DE but also improves the case that vectors with a distinct trend are assigned to the same class sequence, which may provide a powerful aid to feature extraction process of FSDE [31,32]. The calculation equation for factor *f* is as follows:

(20) $$f=\left\lfloor \frac{\max(|d_{y}|)}{\rho \cdot std(|d_{x}|)}\right\rfloor$$

where ⌊⋅⌋ returns the largest integer that is less than or equal to the value in brackets, $d_{x}=\left\{x_{i+1}-x_{i}|i=1,2,\ldots ,n-1\right\}$, $d_{y}=\left\{y_{i+1}-y_{i}|i=1,2,\ldots ,m-1\right\}$, *std* represent the standard deviation and *ρ* is a precision parameter. Obviously, if $\rho >\frac{\max(|d_{y}|)}{std(|d_{x}|)}$, the value of *f* can only be 0, and this additional element will not affect the results of dispersion pattern, which means that the value of FSDE will be exactly the same as original DE. If $\rho \in (0,\frac{\max(|d_{y}|)}{std(|d_{x}|)}]$, *f* will have more possible values, the closer *ρ* gets to 0, the original dispersion pattern will be subdivided into more new dispersion patterns. As an example, time series [0.5114, 0.5131, 05134, 0.5130], [0.5012, 0.5085, 0.5152, 0.5212] and [0.5326, 0.5249, 0.5158, 0.5072] are constructed with *τ* = 1, *m* = 4, *ρ* = 1, *c* = 6. These time series first map to class sequences according to the mapping strategy of DE, i.e., [4, 4, 4, 4], [4, 4, 4, 4], and [4, 4, 4, 4]. It can be seen that all the time series are mapped to the same dispersion pattern, although their trends are different. Meanwhile, the time series are mapped by the proposed FSDE, factor f is added as an additional element to the end of the class sequence, the new class sequences are [4, 4, 4, 4, 0], [4, 4, 4, 4, 1], and [4, 4, 4, 4, 2]. In the example, the time series originally mapped to the same dispersion pattern are finely mapped to more dispersion patterns. Figure 2 illustrates the way that *f* is added to the sequence $z_{j}^{m,c}$ as an additional element.

After obtaining the new class sequence $z_{j}^{m,c^{\prime }}$, ${\pi_{v_{0}v_{1}\ldots v_{m-1}}}^{\prime }$ denotes a dispersion pattern corresponding to a $z_{j}^{m,c^{\prime }}$. The relative frequency of new dispersion pattern is calculated as:

(21) $$p^{\prime }=\frac{Number\{j|j\leq n-(m-1)\tau ,{\pi_{v_{0}v_{1}\ldots v_{m-1}}}^{\prime }\}}{n-(m-1)\tau }$$

Then, similar to the definition of dispersion entropy, the proposed fine-sorted dispersion entropy can be defined as:

(22) $$FSDE(x,m,c,\tau ,f)=-\sum_{\pi =1}^{c^{m}}p^{\prime }\cdot \ln(p^{\prime })$$

### 3.2. Mutation SCA-PSO Optimization

#### 3.2.1. Sine Cosine Algorithm

Sine cosine algorithm (SCA) is a new swarm optimization algorithm, which is simple in structure and easy to realize. Using only sine and cosine functions, SCA achieves a good optimization effect. Exploration and exploitation are the two key phases when SCA processes optimization a problem (see [26] for more details). With an elaborate mechanism of exploration and exploitation, SCA is able to search for feasible solutions quickly in the searching space. Let *Z_i_* = (*Z_i_*_l_, *Z_i_*_2_, …, *Z_id_*)*^T^* be the position of *i*-th individual, whereupon each solution of the optimization problem corresponds to a position of a corresponding individual in the searching space, where *d* is the dimension of individuals. The core updating equation of SCA is as follows:

(23) $$Z_{i}(l+1)=\left\{ \begin{array}{l} Z_{i}(l)+r_{1}\times \sin(r_{2})\times |r_{3}P_{i}(l)-Z_{i}(l)|,\;r_{4}<0.5\\ Z_{i}(l)+r_{1}\times \cos(r_{2})\times |r_{3}P_{i}(l)-Z_{i}(l)|,\;r_{4}\geq 0.5 \end{array}\right.$$

where *P_i_* = (*P_i_*_l_, *P_i_*_2_, …, *P_id_*)*^T^* is the best position of individual *i*. The parameter *r*_1_ = *a* − *l*(*a*/*l*_max_) determine the region of individual *i* at next iteration, where *l*_max_, *l*, and *a* are the maximum number of iterations, the current number of iterations, and a constant, respectively. The parameter *r*_2_ is a random number in the scope of [0, 2π], which defines the distance that the next movement of individuals should be towards. To stochastically emphasize (*r*_3_ > 1) or deemphasize (*r*_3_ < 1) the effect of the best position searched so far, a random weight *r*_3_ within [0, 2] is brought into the equation. Finally, the parameter *r*_4_ is a random number in the range of [0, 1] to switch fairly between sine and cosine components.

#### 3.2.2. Particle Swarm Optimization

Particle swarm optimization (PSO) is a classical stochastic optimization algorithm based on swarm intelligence. In the PSO algorithm, each particle corresponds to a potential solution of the problem to be optimized [33]. Particle *P_i_* has its own velocity and position, which are expressed as *d*-dimensional vectors *v_i_* = (*v_i_*_l_, *v_i_*_2_, …, *v_id_*)*^T^* and *s_i_* = (*s_i_*_l_, *s_i_*_2_, …, *s_id_*)*^T^*, respectively. Particle *P_i_* keeps track of the optimal solution it has found so far, namely, individual extreme value *P_i_^best^*. The optimal solution found by the whole population is called the global extreme value *P_i_^gbest^*. Particles of the swarm update themselves by tracking the above two extremes values. PSO generates a group of particles randomly to initiate the optimization and then solves the problem by iterations. In the *l*-th iteration, particle *P_i_* updates its velocity and position according to the following two equations:

(24) $$v_{i}(l+1)=w\cdot v_{i}(l)+c_{1}\cdot rand(P_{i}^{best}-P_{i}(l))+c_{2}\cdot rand(P_{i}^{gbest}-P_{i}(l))$$

(25) $$s_{i}(l+1)=s_{i}(l)+v_{i}(l)$$

where *w* is the inertia weight which is employed to balance the global and local search ability of the algorithm. *c*_1_ and *c*_2_ are learning factors, *rand* is a random number between (0, 1), *l* is the current number of iterations.

#### 3.2.3. Mutation SCA-PSO Optimization

In the updating strategy of SCA, sine and cosine functions in Equation (23) possess the outstanding ability of exploration but also have some drawbacks, such as slow convergence and the likelihood of being trapped in a local optimum. The reason for the above problems is that parameters of SCA are difficult to set properly, so a poor combination of parameters will result in weak exploitation, but its exploration phase will not be affected [34]. On the other hand, the PSO algorithm has the advantages of information exchange among particles and good robustness, which lead to the high probability of exploiting local optimal solution. However, similar to SCA, PSO also has the problem of local optimum in the later stage of iterations.

**Figure 3:**
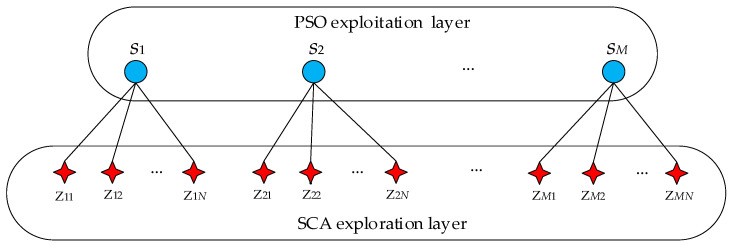
The structure of the mutation sine cosine algorithm and particle swarm optimization (SCA-PSO).

Since SCA and PSO have their own strengths, the combination of capabilities between SCA and PSO is expected to improve the convergence performance, which may make the hybrid algorithm powerful in both exploring different regions and exploiting promising regions. As shown in Figure 3, the SCA-PSO approach is constructed in a hierarchical form. The top layer contains *M* particles which are searching solutions according to the strategy of PSO. The bottom layer contains *M·N* individuals which are evenly distributed to each top layer particle (total *M* groups) and their positions update by SCA strategy.

In iterations, the optimal value found by each group in the bottom layer is saved by a top layer particle. Through this operation, the top layer particles focus on exploitation by using advantages of PSO, while the bottom layer individuals focus on exploration, giving play to advantages of SCA [34]. The updating strategy of bottom layer individuals is changed to the following equation:

(26) $${Z_{ij}}^{l+1}=\left\{ \begin{array}{l} {Z_{ij}}^{l}+r_{1}\times \sin(r_{2})\times |r_{3}{s_{i}}^{l}-{Z_{ij}}^{l}|,\;r_{4}<0.5\\ {Z_{ij}}^{l}+r_{1}\times \cos(r_{2})\times |r_{3}{s_{i}}^{l}-{Z_{ij}}^{l}|,\;r_{4}\geq 0.5 \end{array}\right.$$

where *Z_ij_^l^* is the position of *j*-th (*j* = 1, 2, …, *N*) bottom layer individual that belongs to the *i*-th (*i* = 1, 2, …, *M*) top layer particle. The position of *i*-th top layer particle is described as *s_i_^l^*. *l* is the current number of iterations.

Moreover, analysis of PSO shows that the population diversity of PSO gradually decreasing with each iteration and it is more likely to result in a local optimum in the later stage of iterations. To solve this problem, a mutation operator is introduced to enrich the diversity of PSO particles [35]. The updating rules of top layer particles containing the mutation operators are as follows:

(27) $$v_{i}^{l}=w\cdot v_{i}^{l}+c_{1}\cdot rand(s_{i}^{best}-s_{i}^{l})+c_{2}\cdot rand(s^{gbest}-s_{i}^{l})$$

(28) $${s_{i}}^{l+1}=\left\{ \begin{array}{ll} {s_{i}}^{l}+{v_{i}}^{l} & ,l\neq nT\\ {s_{i}}^{l}[1+G(0.5-unif)] & ,l=nT \end{array}\right.n=1,2,\ldots$$

where *G* and *T* (*T* < *l*_max_) are the given mutation amplitude and mutation period, respectively, while *unif* represents a random number that conforms to the uniform distribution *U* (0, 1). It can be observed that during the updating process, the position *s_i_* will mutate periodically, which gives PSO particles a certain ability to jump out of the local optimum even in the later stage of iterations.

The computational complexity of the proposed mutation sine cosine algorithm and particle swarm optimization (SCA-PSO) algorithm is O(lM(Ntsca+tpso)), where *t*_sca_ and *t*_pso_ are the computational costs of updating all the search agents in each iteration for SCA and PSO, respectively. Accordingly, the computational complexity of SCA and PSO are *O*(*lnt*_sca_) and *O*(*lnt*_pso_), respectively.

**Figure 4:**
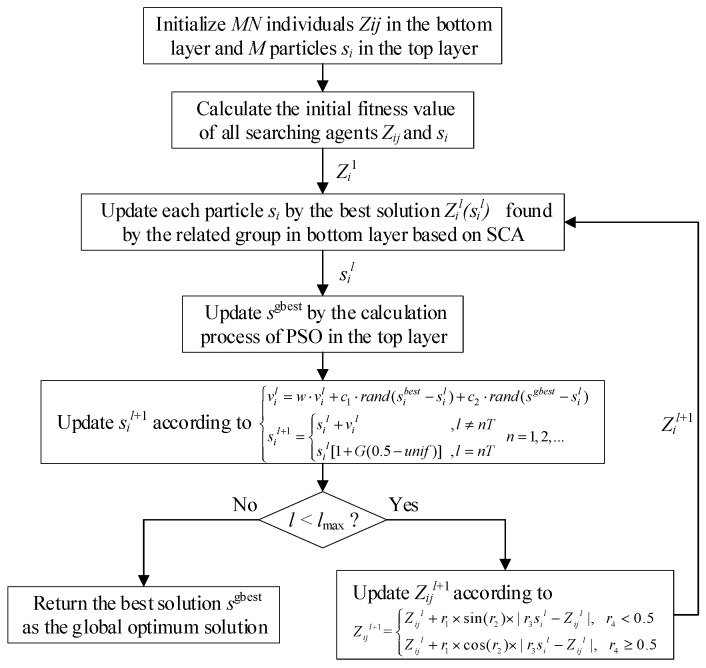
Block diagram of the proposed mutation operator, sine cosine algorithm and particle swarm optimization (MSCAPSO).

The main steps of proposed mutation SCA-PSO (MSCAPSO) approach are as below, and its block diagram is shown in Figure 4:

*Step* 1: Initialize *M* particles in the top layer as well as *M·N* individuals in the bottom layer randomly and set mutation parameters;

*Step* 2: Calculate the fitness values of all searching agents;

*Step* 3: Update *s_i_* on the basis of the best solution found by each group in the bottom layer according to Equation (26);

*Step* 4: Update *s_i_^best^* in top layer based on Equations (27) and (28);

*Step* 5: If the maximum number of iterations is not meet, go to *step* 3;

*Step* 6: Return the value of *s_i_^gbest^* as the optimal solution.

### 3.3. Fault Diagnosis by FSDE and MSCAPSO Optimized SVM

In this section, a novel fault diagnosis method based on FSDE and SVM optimized by MSCAPSO is proposed to improve the fault diagnosis accuracy. The specific steps are detailed as follows:

*Step* 1: Determine the mode number *K* of VMD by central frequency observation method;

*Step* 2: Decompose samples into sets of IMFs with VMD;

*Step* 3: Calculate the FSDE value of each IMF;

*Step* 4: Construct feature vectors of different fault samples with FSDE values;

*Step* 5: Optimize parameters *C* and *g* for SVM with the proposed MSCAPSO optimization method;

*Step* 6: Train the SVM model with optimal parameters *C* and *g*;

*Step* 7: Apply the optimal SVM model to classify different fault samples and evaluate the performance of the model.

**Figure 5:**
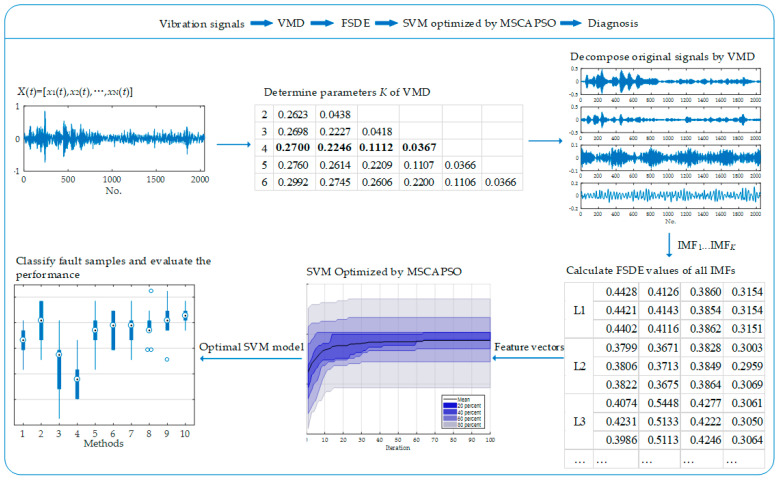
Flowchart of the proposed fault diagnosis method.

The flowchart of the proposed fault diagnosis model is shown in Figure 5.

## 4. Engineering Application

### 4.1. Data Collection

Vibration signals with different fault locations and sizes gathered from Bearings Data Center of Case Western Reserve University (CWRU) [36] were employed as the experiment data in this research. The experiment stand was mainly composed of a motor, an accelerometer, a torque sensor/encoder and a dynamometer. The bearing was a deep groove ball bearing with model SKF6205-2RS. Accelerometers were applied to collect vibration signal. Meanwhile, the torque sensor/encoder was for the measurement of power and speed. By using electro-discharge machining, the experiment device simulated three fault states of the rolling bearing: inner race fault, ball element fault, and outer race fault, and the depth of faults was 0.011 inches. In the experiment, vibration signals collected from the drive end were taken as the research objects, where the sample frequency was 12 kHz. To fully verify the effectiveness of the proposed fault diagnosis method, nine types samples were applied in this paper, namely inner race fault, ball fault, and outer race fault with diameters of 0.007, 0.014, and 0.021 inches. In addition, the experiments are conducted under the load of 2, 1, and 0 hp with motor speed 1750, 1772, and 1797 rpm. Further, the vibration signal of each type of faults contained 59 samples, and each sample contained 2048 sample points. The experimental data are listed in Table 1.

### 4.2. Application to Fault Diagnosis of Rolling Bearings

The proposed FSDE-MSCAPSO method has been applied to diagnose nine types of faults and compared with other six relevant fault diagnosis methods at feature extraction as well as parameter optimization stage. More specifically, fuzzy entropy (FE) [37], permutation entropy (PE) [4], and dispersion entropy (DE) were applied at the feature extraction stage for comparison; SCA and PSO were applied at the parameter optimization stage. The parameters of the contrastive experiments were set in the same way as the proposed method.

**Figure 6:**
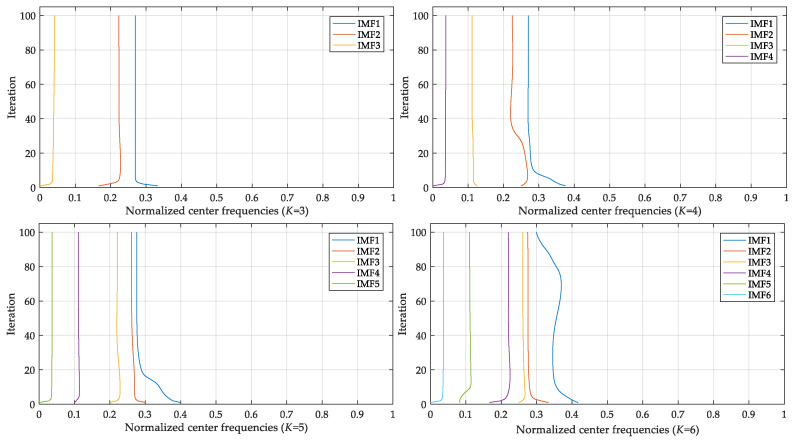
The variation of central frequency with iteration under different *K* values.

In the proposed method, the first step is to decompose the signal of each type of fault into a set of IMFs. The mode number *K* of VMD needs to be determined in advance with the central frequency observation method. If *K* is too large, central frequencies of neighboring IMFs may be too close, then mode mixing will emerge. Normalized central frequencies under different *K* values are listed in Table 2, where *K* was ascertained by the sample of ball fault with a diameter of 0.014 inches (L5). As listed in Table 2, when *K* was set 5, the first two central frequencies were relatively close. Similarly, as illustrated in Figure 6, during the iterative calculation of VMD, if *K* was 5 or greater, there were some central frequencies of IMFs close to each other, which meant excessive decomposition had occurred. Hence, parameter *K* was set to 4.

**Figure 7:**
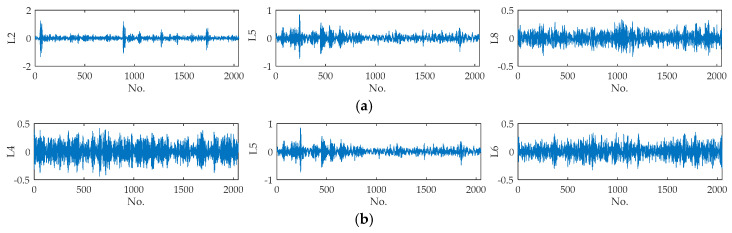
Time domain waveforms of fault signals: (**a**) signals with different fault positions (L2, L5, L8), (**b**) signals with different defect sizes (L4, L5, L6).

**Figure 8:**
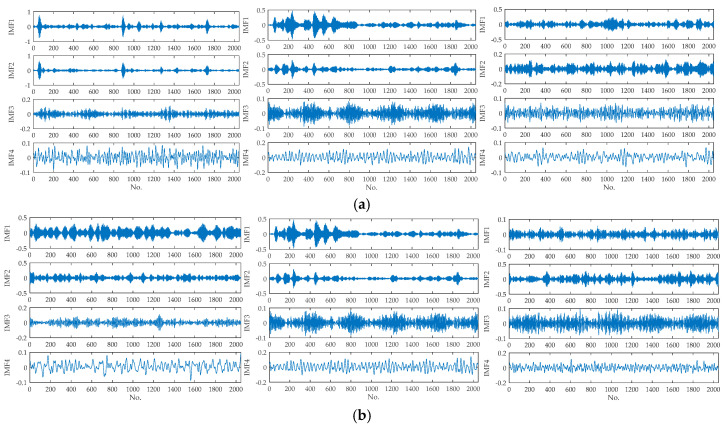
The variational mode decomposition (VMD) results of fault signals: (**a**) decomposition results with different fault positions (L2, L5, L8), (**b**) decomposition results with different defect sizes (L4, L5, L6).

The waveforms of the original signals are shown in Figure 7. It can be observed that waveforms of different fault positions or different defect sizes are different to a certain extent. The examples of decomposition results with different fault positions (L2, L5, L8) and different defect sizes (L4, L5, L6) are illustrated in Figure 8, which shows that IMFs decomposed from the same types of faults are quite different.

When the IMFs of all samples were obtained, FSDE values were calculated to construct fault feature vectors, during which four parameters needed to be chosen properly, namely, embedding dimension *m*, number of class *c*, time delay *τ*, and precision parameter *ρ*. Theoretically, *m* is usually set from two to seven, if *m* is large, there will be a high computational cost. When dealing with *c*, it is usually suggested to choose from four to eight, if *c* is small, it may not be able to recognize two values with very different amplitudes [38]. *ρ* is the key parameter that distinguishes FSDE from DE. The closer *ρ* to 0, an original dispersion pattern will be subdivided into more new dispersion patterns, however, FSDE may also be oversensitive to small changes and increase the computation. Considering all these factors, embedding dimension *m* was set at three and the number of class *c* was set at six in our research. Moreover, for the practical purpose, it was recommended to assume the time delay *τ* = 1 [38], while precision parameter *ρ* was set as 2.9 to balance the precision and computational complexity.

**Figure 9:**
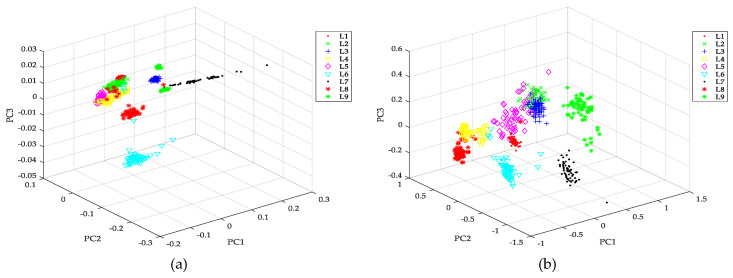

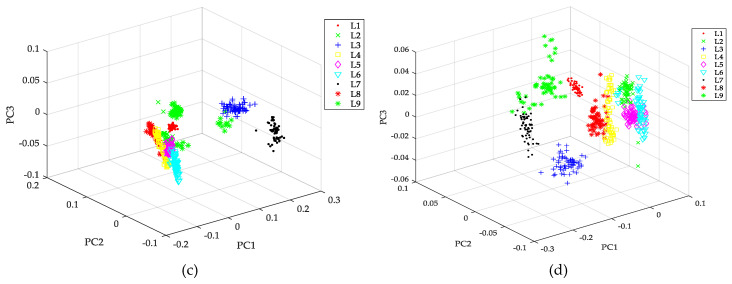
3D projection of entropy vectors for different types of faults: (**a**) vectors of permutation entropy, (**b**) fuzzy entropy, (**c**) dispersion entropy(**d**), fine-sorted dispersion entropy.

The first three FSDE and DE vectors of different fault samples (L1–L9) are compared in Table 3, which shows that FSDE value of every IMF is different from the corresponding DE value, but the overall trend remains similar. 3D projection of entropy vectors for different types of faults is shown in Figure 9. It can be seen that feature vectors extracted by PE are not clearly distinguished, quite a lot of feature vectors from different types of faults are still mixed together. This situation is improved in the figures of FE and DE to a certain extent, while feature vectors extracted by FSDE are basically well distinguished.

In the optimization stage, the feature vectors from different types of faults were divided randomly into two parts with 30 and 29 vectors, where 30 were used for training while the rest, 29, for testing. The proposed MSCAPSO approach was applied to optimize the penalty factor *C* and the kernel parameter *g* of SVM. The total number of searching agents and iteration times were set 40 and 100, respectively, while searching ranges of *C* and *g* were both [2^−10^, 2^10^]. Considering the maximum number of iterations and the number of optimization parameters, the mutation period was set at five and mutation amplitude was set at one. Fitness values were calculated by five-fold cross-validation for the training samples. Hence, the optimization effect of the given *C* and *g* could be measured by the average accuracy of cross-validation, then the optimal *C* and *g* were obtained.

**Figure 10:**
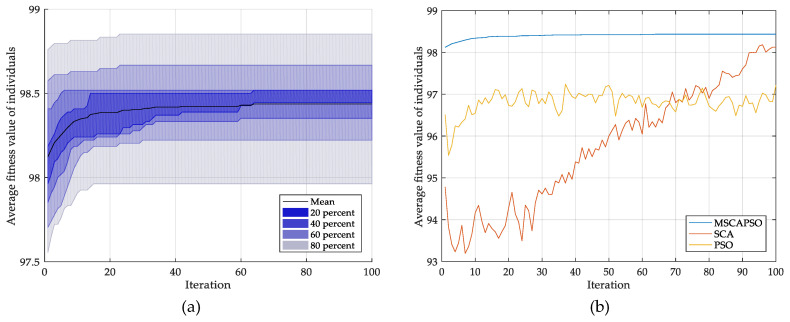
Convergence procedure of proposed optimization approach: (**a**) distribution of convergence curves in 10 runs (**b**) comparison of different optimization methods.

The convergence procedure of MSCAPSO is depicted in Figure 10a, from which it can be observed that the average fitness value of particles rises rapidly at an early stage of the iterations and then tends to be stable. That is, the particles are close to the global optimal solution. The shaded part shows the distribution of convergence curves in 10 runs. The comparison of different optimization methods is shown in Figure 10b, where all convergence experiments are based on the same FSDE feature vectors, and each convergence curve is the average of 10 runs. The figure shows that the convergence curve of PSO fluctuates greatly, and the average fitness value is difficult to be further improved after 50 iterations. SCA has a good overall convergence trend, but the convergence rate is significantly lower than the other two methods. MSCAPSO has already converged to a high level in the early stage and keeps the best convergence effect among all methods in the whole iterations.

With the optimal parameters *C* and *g*, the SVM model optimized by MSCAPSO was trained and applied to recognize testing samples. For an in-depth verification of the proposed method, diagnosis results were averaged over 10 runs, and training samples were chosen at random in every run. Furthermore, the deviation of each result was calculated as a reference for result evaluation. In this application, adjusted Rand index (ARI), normalized mutual information (NMI), F-measure (F), and accuracy (ACC) as four widely used evaluation metrics are employed to evaluate the diagnosis results [39,40], which reflects the matching degree between classification result and real distribution information of samples. The scope of ARI is [−1, 1] and the rest scopes are all [0, 1]. A higher value indicates a better classification performance. The optimal *C* and *g* are presented corresponding to the best accuracy among 10 runs.

**Figure 11:**
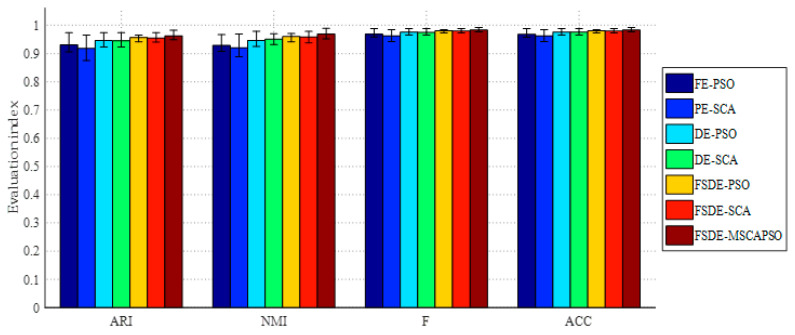
Comparison of evaluation values for different methods (2 hp).

Fault diagnosis results with different methods under variable load conditions are shown in Table 4 and Figure 11. It can be observed from Table 4 that four evaluation metrics of the proposed FSDE-MSCAPSO method are all the highest when the motor loads are 2 hp and 1 hp, i.e., 0.9637, 0.9646, 0.9839, 0.9839 and 0.9615, 0.9598, 0.9827, 0.9828. As well, the deviations of the evaluation values are also at a very small level among all methods. When the motor load is 0 hp, although the value of proposed method is slightly lower than FE-PSO in each metric, its performance is still better than DE-PSO/SCA methods than before improvements. To be specific, the comparison of FE-PSO, DE-PSO and FSDE-PSO indicates that the proposed FSDE has a better feature extraction ability than FE, PE and DE in the field of fault diagnosis. Similarly, this fact is also verified in the comparison among PE-SCA, DE-SCA, and FSDE-SCA. Moreover, it can be concluded from the contrastive experiments between FSDE-SCA/PSO and FSDE-MSCAPSO that the proposed MSCAPSO optimization strategy improves the diagnosis performance of SVM.

**Figure 12:**
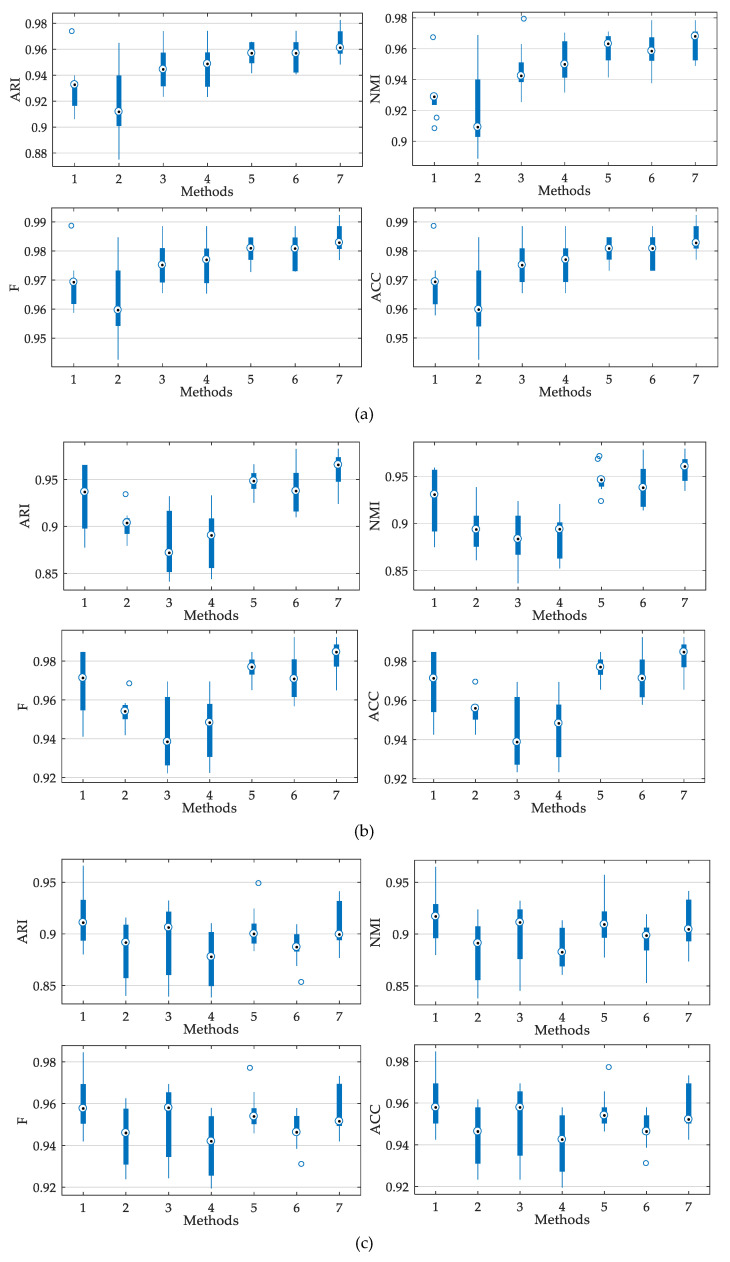
Boxplots of evaluation values for different methods under variable load conditions: (**a**) 2hp, (**b**) 2hp, (**c**) 0hp; the x-axis tick labels correspond to: 1: Fuzzy entropy-particle swarm optimization (FE-PSO), 2: Permutation entropy-sine cosine algorithm (PE-SCA), 3: Dispersion entropy-particle swarm optimization (DE-PSO), 4 Dispersion entropy-sine cosine algorithm (DE-SCA), 5: Fine-sorted dispersion entropy-particle swarm optimization (FSDE-PSO), 6: Fine-sorted dispersion entropy-sine cosine algorithm (FSDE-SCA), 7: Fine-sorted dispersion entropy-mutation operator, sine cosine algorithm and particle swarm optimization (FSDE-MSCAPSO).

**Figure 13:**
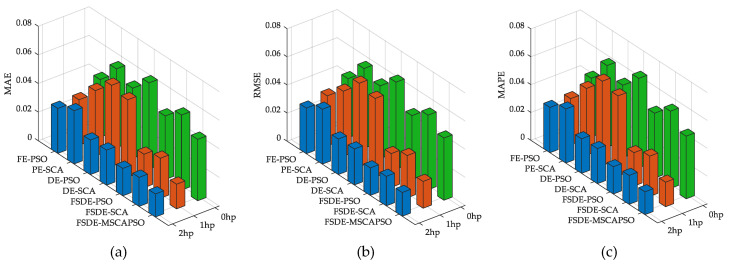
Comparison of mean absolute error (MAE), root mean square error (RMSE), and mean absolute percentage error (MAPE) for diagnosis models under different load conditions: (**a**) MAE, (**b**) RMSE, (**c**) MAPE.

The boxplots of evaluation values are shown in Figure 12, illustrating the performances of different diagnostic methods. As shown in the figure, the proposed method achieves better overall performance and stability for fault diagnosis than other contrastive methods. Furthermore, several statistical metrics that have been widely used for robustness evaluation [10,41,42] are applied to demonstrate the robustness of the diagnosis models, i.e., mean absolute error (MAE), root mean square error (RMSE), and mean absolute percentage error (MAPE). The error metrics of the outputs of different models under various loads are listed in Table 5, as well as the corresponding histograms shown in Figure 13. It can be observed that error metrics of FSDE-MSCAPSO are the lowest when the motor loads are 2 hp and 1 hp. When the motor load is 0 hp, although the metric values of the proposed method are not the lowest among all models, it is still at a low level and in the second position in each metric, indicating the proposed model has good robustness on the whole.

## 5. Discussion

After the detailed comparative analysis above, the superiority of the proposed FSDE and MSCAPSO methods have been effectively demonstrated, as well, the accuracy of the diagnosis model has also been verified by experiments on various loads, fault sizes, and locations. It is worth noting that the proposed FSDE method only considers the relationship between neighboring elements in a given series, and the overall information of the series is not taken into account. According to the previous references [31,43], the improved method using the overall amplitude information of the series has been realized on other entropy methods, which is expected to further improve the performance of the proposed method. Furthermore, some of the parameters in the FSDE are still empirical values in the present paper. In future studies, reasonable metrics can be introduced to evaluate the influence of parameters on the approximation between FSDE and DE, so as to better determine the value of parameters. Furthermore, the proposed MSCAPSO algorithm can be optimized through better strategies to further improve the convergence speed and global search ability. In addition, the multi-objective optimization that has been widely utilized in the field of controlling [44,45,46,47] could be implemented in fault diagnosis, which is expected to improve the accuracy and reduce the variance of the outputs of the model [48]. Moreover, although the diagnosis experiments considered various load conditions, fault sizes, and locations, the signal to noise ratio of the CWRU bearing data is relatively high, and the conclusions obtained in the experiment may not be very comprehensive. In the future work, we plan to build our own bearing test device to collect many more vibration signals, and the results obtained under such conditions may be more practical.

Considering that the CWRU bearing data set has been selected by many researchers as experimental data to validate their methods and techniques, the diagnosis accuracies of some other methods based on this data set are listed in Table 6 to compare with the methods presented in this paper. Note that the segmentation or fault types of experimental samples may not be exactly the same, the accuracies listed is for general description.

## 6. Conclusions

A novel fault diagnosis method for rolling bearings by fine-sorted dispersion entropy and mutation SCA-PSO optimized SVM is presented to diagnose faults of various sizes, locations, and motor loads in this study. Due to the non-stationarity of original vibration signals, signals collected from different types of faults were firstly split by VMD into sets of IMFs, before which the decomposing mode number *K* was determined by a central frequency observation method. Then an improved dispersion entropy containing an additional factor, termed FSDE, was proposed to enhance the perception for relationship information between neighboring elements. It was employed to construct the feature vectors of different fault samples later. Afterward, a hybrid optimization strategy combining advantages of mutation operator, SCA and PSO called MSCAPSO, was proposed to optimize the SVM model. The optimal SVM model was subsequently applied to realize the classification for different fault samples. The contrastive experiments in Section 4 were implemented among the proposed FSDE-MSCAPSO method and other relevant methods, where nine types of faults with different locations and sizes were successfully diagnosed. The analysis of results indicates that the proposed method achieves the highest 0.9637, 0.9646, 0.9839, and 0.9839 in four widely used evaluation metrics, which exhibits its superior precision and stability over other methods.

## Figures and Tables

**Table 1:** Description of the experimental data.

Motor Load (hp)	Position of Fault	Defect Size (Inches)	Label of Classes	Number of Samples
2/1/0	Inner race	0.007	L1	59
	Inner race	0.014	L2	59
	Inner race	0.021	L3	59
	Ball	0.007	L4	59
	Ball	0.014	L5	59
	Ball	0.021	L6	59
	Outer race	0.007	L7	59
	Outer race	0.014	L8	59
	Outer race	0.021	L9	59

**Table 2:** Normalized central frequencies with different *K* values.

Number of Modes	Normalized Central Frequencies							
2	0.2623	0.0438						
3	0.2698	0.2227	0.0418					
4	0.2700	0.2246	0.1112	0.0367				
5	0.2760	0.2614	0.2209	0.1107	0.0366			
6	0.2992	0.2745	0.2606	0.2200	0.1106	0.0366		
7	0.3301	0.2754	0.2610	0.2206	0.1146	0.0548	0.0315	
8	0.3663	0.2847	0.2711	0.2548	0.2180	0.1144	0.0546	0.0314

**Table 3:** Fine-sorted dispersion entropy and dispersion entropy of intrinsic mode functions (IMFs).

Fault Label	Sample Number	Fine-Sorted Dispersion Entropy				Dispersion Entropy			
		IMF1	IMF2	IMF3	IMF4	IMF1	IMF2	IMF3	IMF4
L1	1	0.4428	0.4126	0.3860	0.3154	0.4110	0.3740	0.3202	0.2509
	2	0.4421	0.4143	0.3854	0.3154	0.4074	0.3734	0.3204	0.2520
	3	0.4402	0.4116	0.3862	0.3151	0.4030	0.3700	0.3201	0.2495
L2	1	0.3799	0.3671	0.3828	0.3003	0.3486	0.3344	0.3157	0.2370
	2	0.3806	0.3713	0.3849	0.2959	0.3606	0.3520	0.3170	0.2380
	3	0.3822	0.3675	0.3864	0.3069	0.3611	0.3347	0.3163	0.2452
L3	1	0.4074	0.5448	0.4277	0.3061	0.3730	0.5225	0.3881	0.2514
	2	0.4231	0.5133	0.4222	0.3050	0.3907	0.4924	0.3829	0.2519
	3	0.3986	0.5113	0.4246	0.3064	0.3576	0.4859	0.3773	0.2509
L4	1	0.3993	0.3838	0.3611	0.2623	0.3146	0.3056	0.2970	0.2031
	2	0.4034	0.3876	0.3555	0.2452	0.3120	0.3041	0.2914	0.1789
	3	0.4028	0.3874	0.3622	0.2891	0.3135	0.3108	0.2968	0.2288
L5	1	0.3708	0.3707	0.3884	0.2769	0.3063	0.3112	0.3176	0.2218
	2	0.3638	0.3725	0.3861	0.2799	0.3043	0.3129	0.3143	0.2198
	3	0.3731	0.3856	0.3873	0.2783	0.3021	0.3203	0.3162	0.2206
L6	1	0.3513	0.3916	0.3938	0.3026	0.2897	0.3157	0.3190	0.2397
	2	0.3847	0.3943	0.3944	0.3031	0.3109	0.3153	0.3178	0.2415
	3	0.3851	0.3882	0.4021	0.3127	0.3117	0.3135	0.3187	0.2499
L7	1	0.4561	0.5815	0.4964	0.3546	0.4372	0.5582	0.4718	0.2926
	2	0.4613	0.5820	0.4910	0.3444	0.4414	0.5569	0.4685	0.2859
	3	0.4606	0.5864	0.4960	0.3754	0.4395	0.5632	0.4736	0.3167
L8	1	0.4052	0.3892	0.3477	0.2739	0.3282	0.3083	0.2825	0.2159
	2	0.4162	0.3953	0.3324	0.2770	0.3329	0.3159	0.2669	0.2165
	3	0.4024	0.3884	0.3539	0.2979	0.3296	0.3140	0.2892	0.2320
L9	1	0.4793	0.4115	0.3643	0.3088	0.4746	0.4041	0.3244	0.2656
	2	0.4899	0.3863	0.3996	0.3169	0.4855	0.3758	0.3743	0.2778
	3	0.4906	0.5040	0.4291	0.3139	0.4825	0.4966	0.4234	0.2751

**Table 4:** Fault diagnosis results with different methods under variable load conditions.

Motor Load (hp)	Methods	*C*	*g*	Evaluation Metrics			
				Adjusted Rand Index (ARI)	Normalized Mutual Information (NMI)	F-Measure (F)	Accuracy (ACC)
2	FE-PSO	204.766	7.978	0.9309 [−0.025, 0.043]	0.9288 [−0.021, 0.038]	0.9687 [−0.010, 0.020]	0.9686 [−0.011, 0.020]
	PE-SCA	616.339	411.111	0.9185 [−0.043, 0.046]	0.9207 [−0.032, 0.048]	0.9628 [−0.020, 0.022]	0.9628 [−0.020, 0.022]
	DE-PSO	13.124	1024	0.9461 [−0.023, 0.028]	0.9463 [−0.021, 0.033]	0.9759 [−0.010, 0.013]	0.9759 [−0.010, 0.013]
	DE-SCA	12.762	420.762	0.9456 [−0.022, 0.028]	0.9513 [−0.020, 0.019]	0.9755 [−0.010, 0.013]	0.9755 [−0.010, 0.013]
	FSDE-PSO	54.370	151.024	0.9572 [−0.016, 0.009]	0.9603 [−0.019, 0.011]	0.9808 [−0.008, 0.004]	0.9808 [−0.008, 0.004]
	FSDE-SCA	25.206	200.267	0.9549 [−0.014, 0.019]	0.9589 [−0.021, 0.019]	0.9797 [−0.007, 0.009]	0.9797 [−0.007, 0.009]
	FSDE-MSCAPSO	6.085	390.627	0.9637 [−0.019, 0.019]	0.9646 [−0.016, 0.014]	0.9839 [−0.007, 0.008]	0.9839 [−0.007, 0.008]
1	FE-PSO	6.908	2.358	0.9304 [−0.053, 0.035]	0.9243 [−0.050, 0.035]	0.9681 [−0.027, 0.017]	0.9682 [−0.026, 0.016]
	PE-SCA	69.617	294.358	0.9019 [−0.023, 0.032]	0.8942 [−0.0433, 0.044]	0.9537 [−0.012, 0.015]	0.9544 [−0.012, 0.015]
	DE-PSO	2.217	586.466	0.8814 [−0.040, 0.051]	0.8827 [−0.046, 0.041]	0.9429 [−0.021, 0.027]	0.9433 [−0.020, 0.026]
	DE-SCA	331.824	250.205	0.8864 [−0.042, 0.047]	0.8869 [−0.035, 0.034]	0.9458 [−0.023, 0.024]	0.9460 [−0.023, 0.023]
	FSDE-PSO	2.754	1024.000	0.9483 [−0.023, 0.018]	0.9468 [−0.024, 0.024]	0.9765 [−0.011, 0.008]	0.9766 [−0.011, 0.008]
	FSDE-SCA	4.532	690.248	0.9386 [−0.029, 0.044]	0.9387 [−0.025, 0.040]	0.9718 [−0.015, 0.021]	0.9720 [−0.014, 0.020]
	FSDE-MSCAPSO	5.004	1024.000	0.9615 [−0.038, 0.021]	0.9598 [−0.025, 0.019]	0.9827 [−0.018, 0.010]	0.9828 [−0.017, 0.010]
0	FE-PSO	9.453	9.781	0.9141 [−0.034, 0.052]	0.9144 [−0.035, 0.051]	0.9596 [−0.018, 0.025]	0.9598 [−0.017, 0.025]
	PE-SCA	853.002	32.932	0.8850 [−0.045, 0.031]	0.8862 [−0.048, 0.038]	0.9447 [−0.021, 0.018]	0.9448 [−0.021, 0.017]
	DE-PSO	16.319	456.170	0.8942 [−0.055, 0.038]	0.8997 [−0.054, 0.032]	0.9512 [−0.027, 0.018]	0.9510 [−0.028, 0.018]
	DE-SCA	155.860	163.498	0.8753 [−0.037, 0.035]	0.8855 [−0.025, 0.028]	0.9396 [−0.020, 0.018]	0.9398 [−0.020, 0.018]
	FSDE-PSO	16.138	549.663	0.9036 [−0.020, 0.045]	0.9111 [−0.034, 0.046]	0.9554 [−0.010, 0.022]	0.9556 [−0.009, 0.021]
	FSDE-SCA	1024.000	2.400	0.8873 [−0.034, 0.022]	0.8948 [−0.042, 0.024]	0.9473 [−0.016, 0.011]	0.9475 [−0.016, 0.010]
	FSDE-MSCAPSO	216.051	448.183	0.9079 [−0.031, 0.033]	0.9086 [−0.035, 0.033]	0.9567 [−0.015, 0.017]	0.9571 [−0.015, 0.016]

**Table 5:** Error metrics of results of different methods.

Method	MAE			RMSE			MAPE		
	2 hp	1 hp	0 hp	2 hp	1 hp	0 hp	2 hp	1 hp	0 hp
FE-PSO	0.0314	0.0318	0.0402	0.0324	0.0354	0.0420	0.0325	0.0331	0.0421
PE-SCA	0.0372	0.0456	0.0552	0.0390	0.0462	0.0566	0.0388	0.0478	0.0586
DE-PSO	0.0241	0.0567	0.0490	0.0253	0.0592	0.0516	0.0248	0.0605	0.0519
DE-SCA	0.0245	0.0540	0.0602	0.0256	0.0558	0.0617	0.0252	0.0573	0.0642
FSDE-PSO	0.0192	0.0234	0.0444	0.0195	0.0240	0.0453	0.0195	0.0240	0.0466
FSDE-SCA	0.0203	0.0280	0.0525	0.0210	0.0300	0.0531	0.0208	0.0289	0.0555
FSDE-MSCAPSO	0.0161	0.0172	0.0429	0.0169	0.0190	0.0443	0.0164	0.0176	0.0450

**Table 6:** Comparison among several other fault diagnosis model. The abbreviations are as follows: Chaos quantum sine cosine algorithm (CQSCA), hybrid gravitational search algorithm (HGSA), extreme learning machine (ELM), multivariate autoregressive (MAR), time shift multiscale fuzzy entropy (TSMFE), Laplacian support vector machine (LapSVM), improved multiscale dispersion entropy (IMDE), max-relevance min-redundancy (mRMR), back-propagation neural network (BPNN), improved multiscale permutation entropy (IMPE), quantum behaved particle swarm optimization (QPSO), least squares support vector machine (LSSVM), generalized composite multiscale permutation entropy (GCMPE), semi-supervised kernel Marginal Fisher analysis (SSKMFA).

Reference	Feature Extraction	Classification	Accuracy
[4]	IMFs decomposed by VMD+PE	CQSCA-SVM	0.9789
[8]	IMFs decomposed by EEMD	HGSA-ELM	0.9938
[12]	IMFs decomposed by VMD+MAR	HMGWOSCA-SVM	0.9808
[20]	IMPE	QPSO-LSSVM	0.9800
[22]	TSMFE	C1: LapSVM C2: SVM	C1: 100 C2: 98.44
[49]	IMDE	C1: mRMR+ELM C2: mRMR+BPNN C3: mRMR+SVM	C1: 0.9056 C2:0.8760 C3: 0.8840
[50]	GCMPE	PSO-SVM	0.9889
[51]	SSKMFA	KNN	0.9850

## Nomenclature

**Symbols**		**Abbreviations**	
*K*	mode number	ApEn	approximate entropy
*m_k_*	set of *K* mode functions	ANN	artificial neural network
*ω_k_*	set of central frequencies	ASA	artificial sheep algorithm
*∂_t_*	partial derivative of time *t*	BFA	bacterial foraging algorithm
*δ_t_*	unit pulse function of time *t*	BPNN	back-propagation neural network
*f*(*t*)	real valued input signal	CWRU	Case Western Reserve University
*α*	penalty factor for VMD	CQSCA	chaos quantum sine cosine algorithm
*β*(*t*)	Lagrange multiplier for VMD	DE	dispersion entropy
*w*	weight vector	EMD	empirical mode decomposition
*b*	bias parameter	EEMD	ensemble empirical mode decomposition
*ξ*	slack term	EWT	empirical wavelet transforms
*C*	penalty factor for SVM	ELM	extreme learning machine
*g*	kernel parameter	FE	fuzzy entropy
*μ_i_*	Lagrange multiplier for SVM	FSDE	fine-sorted dispersion entropy
***x***	time series	GCMPE	generalized composite multiscale permutation entropy
*n*	length of time series	HGSA	hybrid gravitational search algorithm
*σ*	variance of the normal distribution	IMPE	improved multiscale permutation entropy
*μ*	expectation of the normal distribution	IMDE	improved multiscale dispersion entropy
$y_{j}^{m}$	reconstruction matrix of ***x***	IMF	intrinsic mode function
*m*	embedding dimension	KNN	k-nearest neighbour
*τ*	time delay	LapSVM	Laplacian support vector machine
*c*	number of class	LSSVM	least squares support vector machine
$z_{j}^{m,c}$	class sequence of DE	MSCAPSO	mutation sine cosine algorithm and particle swarm optimization
$\pi_{v_{0}v_{1}\ldots v_{m-1}}$	dispersion pattern of DE	MHGWOSCA	mutation hybrid grey wolf optimizer and sine cosine algorithm
*f*	factor of FSDE	mRMR	max-relevance min-redundancy
*ρ*	precision parameter	MAR	multivariate autoregressive
$z_{j}^{m,c^{\prime }}$	class sequence of DE	MAE	mean absolute error
${\pi_{v_{0}v_{1}\ldots v_{m-1}}}^{\prime }$	dispersion pattern of FSDE	MAPE	root mean square error
*Z_i_*	position of individual	PSO	particle swarm optimization
*P_i_*	best position of individual	PE	permutation entropy
*v_i_*	velocity of particle	QPSO	quantum behaved particle swarm optimization
*s_i_*	position of particle	RMSE	mean absolute percentage error
*P_i_^best^*	individual extreme value	SCA	sine cosine algorithm
*P_i_^gbest^*	global extreme value	SVM	support vector machine
*c*_1_, *c*_2_	learning factor	SSKMFA	semi-supervised kernel Marginal Fisher analysis
*Z_ij_^l^*	position of bottom layer individual	TSMFE	time shift multiscale fuzzy entropy
*s_i_^l^*	position of top layer particle	VMD	variational mode decomposition
*M*	number of top layer particle		
*N*	number of bottom layer individual		
*G*	mutation amplitude		
*l*	current number of iterations		
